# HLA-DRB1-DQB1 Haplotypes Confer Susceptibility and Resistance to Multiple Sclerosis in Sardinia

**DOI:** 10.1371/journal.pone.0033972

**Published:** 2012-04-11

**Authors:** Eleonora Cocco, Claudia Sardu, Enrico Pieroni, Maria Valentini, Raffaele Murru, Gianna Costa, Stefania Tranquilli, Jessica Frau, Giancarlo Coghe, Nicola Carboni, Matteo Floris, Paolo Contu, Maria Giovanna Marrosu

**Affiliations:** 1 Department Public Health, Clinical and Molecular Medicine, University of Cagliari, Cagliari, Italy; 2 CRS4 (Centro di Ricerca, Sviluppo e Studi Superiori in Sardegna), Science and Technology Park Polaris - Piscina Manna, Pula (Cagliari), Italy; Kyushu University, Japan

## Abstract

**Introduction:**

Genetic predisposition to multiple sclerosis (MS) in Sardinia (Italy) has been associated with five DRB1*-DQB1* haplotypes of the human leukocyte antigen (HLA). Given the complexity of these associations, an in-depth re-analysis was performed with the specific aims of confirming the haplotype associations; establishing the independence of the associated haplotypes; and assessing patients' genotypic risk of developing MS.

**Methods and Results:**

A transmission disequilibrium test (TDT) of the DRB1*-DQB1* haplotypes in 943 trio families, confirmed a higher than expected transmission rate (over-transmission) of the *13:03-*03:01 (OR = 2.9, P = 7.6×10^−3^), *04:05-*03:01 (OR = 2.4, P = 4.4×10^−6^) and *03:01-*02:01 (OR = 2.1, P = 1.0×10^−15^) haplotype. In contrast, the *16:01-*05:02 (OR = 0.5, P = 5.4×10^−11^) and the *15:02-*06:01 (OR = 0.3, P = 1.5×10^−3^) haplotypes exhibited a lower than expected transmission rate (under-transmission). The independence of the transmission of each positively and negatively associated haplotype was confirmed relative to all positively associated haplotypes, and to the negatively associated *16:01-*05:02 haplotype. In patients, carriage of two predisposing haplotypes, or of protective haplotypes, respectively increased or decreased the patient's risk of developing MS. The risk of MS followed a multiplicative model of genotypes, which was, in order of decreasing ORs: *04:05-*0301/*03:01-*02:01 (OR = 4.5); *03:01-*02:01/*03:01-*02:01 (OR = 4.1); and the *16:01-*05:02/*16:01-*0502 (OR = 0.2) genotypes. Analysis of DRB1 and DQB1 protein chain residues showed that the Val/Gly residue at position 86 of the DRB1 chain was the only difference between the protective *16:01- *15:02 alleles and the predisposing *15:01 one. Similarly, the Ala/Val residue at position 38 of the DQB1 chain differentiated the positively associated *06:02 allele and the negatively associated *05:02, *06:01 alleles.

**Conclusions:**

These findings show that the association of specific, independent DRB1*-DQB1* haplotypes confers susceptibility or resistance to MS in the MS-prone Sardinian population. The data also supports a functional role for specific residues of the DRB1 and DQB1 proteins in predisposing patients to MS.

## Introduction

The pathogenesis of multiple sclerosis (MS), the most common disabling disease in young adulthood, is believed to be driven by an inflammatory and degenerative process [1]. Many studies have demonstrated that genetic factors play a role in MS predisposition [2]–[4]. Susceptibility to the disease is conferred by a rather large number of small genetic variants, as recently identified by a genome wide association study [4], with the main genetic determinant located at the human leukocyte antigen (HLA) class II DRB1 and DQB1 loci. Indeed, since the 1972, MS has consistently been associated with the Major Histocompatibility (MHC) region [5]. Successive studies progressively refined the association to the DR2 allele [6] and to specific molecular variants [7], notably the HLA-DRB1*15 (*DRB1-15:01-DQA1*01:02-DQB1*06:02) haplotype, which represents the main disease risk factor in populations of North European origin [4]. However, several different allelic associations are present in South European populations [8]–[10] and in Israel [11], and other secondary DRB1* allelic associations have been found in North European populations [4]. Together, these findings question the idea that only products of molecular variants contained in the *15:01-DQB1*06:02 haplotype are functionally responsible for the autoimmune response to encephalitogenic peptides [12], which causes MS. Moreover, in MS populations of North European ancestry, several studies have determined the presence of alleles conferring resistance and influencing predisposition to the disease [13]–[16]. For instance, the effect of the *15:01 allele, which maximally increases the MS risk in white populations of Northern-European descent [4], is either cancelled by the co-presence of the *14 allele, or is reinforced by the co-presence of the *08 allele [14]–[16]. The high degree of non-random association (linkage disequilibrium, LD) across the MHC region represents an important limit to understanding HLA-disease associations, which may prevent the discrimination of whether an identified variant is the causal gene, or it simply reflects LD. Suggestions that MS risk is influenced by variations both at the DRB1 and the DQB1 region also come from some studies in North European MS patient cohorts [17], [18]. In an attempt to gain insight on the role of HLA class II molecules in inflammatory and demyelinating human diseases such as MS, humanized animal models using transgenic mice have been used. These models are able to express human HLA-DR or –DQ genes, in absence of endogenous mouse class II genes [12], [19]. The role of both DRB1* and DQB1* molecules in susceptibility and resistance to experimental autoimmune encephalomyelitis (EAE) has recently been demonstrated in a HLA double transgenic mouse, thus reinforcing the idea that a synergism between DRB1* and DQB1* genes may contribute to MS predisposition [20].

Sardinia is a major Italian island with a high incidence of MS [21], [22], distinguished by a unique, highly homogeneous genetic make-up, resulting from fixation of alleles and haplotypes that are elsewhere rare or absent [23]. For instance, the *03:01-*02:01 haplotype is extremely frequent, while the *15:01-*06:02 haplotype is only rarely observed in this population [23]. Such genetic differentiation is extremely helpful in defining which HLA alleles and motifs are shared between patients from distantly related populations. A significant positive association with MS and five HLA haplotypes, including the *13:03-*03:01, *04:05-*03:01, *03:01-*02:01, *04:05-*03:02 and *15:01-*06:01 has been reported in the Sardinian population, with different ranges of risk carried by patients/individuals with each associated haplotype [24]. The independence of associated haplotypes was not assessed in the previously mentioned study and, despite the plethora of predisposing HLA molecules, significantly negatively associated haplotypes did not emerge, when displacement effects driven by the positively associated haplotypes were taken into account [24]. Thus, our aim in the present study is to re-analyse the MS risk conferred by HLA class II variants and specifically determined by both DRB1* and DQB1* alleles (DRB1*- DQB1* haplotype) in a more consistent sample of Sardinian MS families. Our intent is to confirm the haplotype associations, establish the independence of the associated haplotypes and assess the risk due to the genotype of Sardinian patients.

## Results

### Analysis of associated HLA-DRB1-DQB1 haplotypes

We first re-analysed the association of DRB1*-DQB1* haplotypes in Sardinian MS patients. For this purpose, transmission disequilibrium test (TDT) analysis was performed in a sample of 943 trio families. Only haplotypes represented in at least 1% of the sample were considered. After correction for the 21 considered haplotypes, three of them were found to be significantly over-transmitted: *13:03-*03:01 (OR = 2.9, 95% CI 1.6–5.4, P = 7.6×10^−3^); *04:05- *03:01 (OR = 2.4, 95% CI 1.7–3.4, P = 4.4×10^−6^); and *03:01- *02:01 (OR = 2.1, 95% CI 1.7–2.4, P = 1.0×10^−5^). Also the *15:01- *06:02 haplotype appeared at first to be over-transmitted (OR = 1.9, 95% CI 1.1–3.3,P = 1.4×10^−2^), but statistical correction greatly reduced the significance (P = 3.0×10^−1^). Finally, two haplotypes resulted as under- transmitted: *16:01-* 05:02 (OR = 0.5, 95% CI 0.4–0.6,P = 5.4×10^−11^) and *15:02- *06:01 (OR = 0.3, 95% CI 0.1–0.5, P = 1.5×10^−3^).

Transmission of the HLA-DRB1*-DQB1* haplotypes to unaffected offspring was also examined in 362 healthy siblings belonging to the 943 families (only one sibling per family was considered in the analysis). Data are reported in Table 1.

**Table 1:** Transmission disequilibrium test in 943 trios multiple sclerosis families.

	Affected offspring								Unaffected offspring							
Haplotype DRB1-DQB1	T	NT	OR	95% C.I.		P	Pc	χ^2^	T	NT	OR	95% C.I.		P	Pc	χ^2^
DRB1*13:03-DQB1*03:01	**40**	**14**	2.9	1.6	5.4	3.6×10^−4^	7.6×10^−3^	12.7	**7**	**11**	0.6	0.2	1.6	3.4×10^−1^	7.2×10^0^	0.7
DRB1*04:05-DQB1*03:01	**115**	**50**	2.4	1.7	3.4	2.1×10^−7^	4.4×10^−6^		**32**	**37**	0.9	0.5	1.4	5.4×10^−1^	1.1×10^1^	1.4
DRB1*08:01-DQB1*04:02	**21**	**9**	2.4	1.1	5.1	2.8×10^−2^	5.8×10^−1^	4.8	**4**	**7**	0.6	0.2	2.0	3.6×10^−1^	7.6×10^0^	2.9
DRB1*03:01-DQB1*02:01	**464**	**264**	2.1	1.7	2.4	4.9×10^−17^	1.0×10^−15^		**127**	**159**	0.7	0.6	1.0	3.2×10^−2^	6.7×10^−1^	3.2
DRB1*15:01-DQB1*06:02	**40**	**21**	1.9	1.1	3.3	1.4×10^−2^	3.0×10^−1^	6.0	**14**	**8**	1.8	0.7	4.2	2.0×10^−1^	4.1×10^0^	0.1
DRB1*04:02-DQB1*03:02	**32**	**18**	1.8	1.0	3.2	4.6×10^−2^	9.7×10^−1^	4.0	**12**	**12**	1.0	0.4	2.2	1.0×10^0^	2.1×10^1^	0.1
DRB1*13:01-DQB1*06:03	**24**	**20**	1.2	0.7	2.2	5.4×10^−1^	1.1×10^1^	0.4	**5**	**11**	0.5	0.2	1.3	1.3×10^−1^	2.8×10^0^	3.6
DRB1*11:04-DQB1*03:01	**118**	**105**	1.1	0.9	1.5	3.7×10^−1^	7.7×10^0^	0.8	**44**	**42**	1.1	0.7	1.6	8.2×10^−1^	1.7×10^1^	0.4
DRB1*04:05-DQB1*03:02	**76**	**69**	1.1	0.8	1.5	5.5×10^−1^	1.2×10^0^	0.4	**36**	**30**	1.2	0.7	2.0	4.5×10^−1^	9.4×10^0^	0.0
DRB1*04:03-DQB1*03:02	**36**	**34**	1.1	0.7	1.7	8.1×10^−1^	1.7×10^1^	0.1	**15**	**14**	1.1	0.5	2.2	8.5×10^−1^	1.8×10^1^	0.5
DRB1*07:01-DQB1*02:01	**73**	**83**	0.9	0.6	1.2	4.1×10^−1^	8.7×10^0^	0.7	**26**	**27**	1.0	0.6	1.7	8.9×10^−1^	1.9×10^1^	0.6
DRB1*12:01-DQB1*03:01	**25**	**31**	0.8	0.5	1.4	4.2×10^−1^	8.8×10^0^	0.7	**13**	**11**	1.2	0.5	2.7	6.8×10^−1^	1.4×10^1^	2.0
DRB1*04:03-DQB1*03:04	**27**	**34**	0.8	0.5	1.3	3.7×10^−1^	7.7×10^0^	0.8	**15**	**11**	1.4	0.6	3.0	4.3×10^−1^	9.0×10^0^	0.9
DRB1*01:01-DQB1*05:01	**68**	**89**	0.8	0.5	1.0	8.6×10^−2^	1.8×10^0^	2.9	**28**	**33**	0.8	0.5	1.4	5.1×10^−1^	1.1×10^1^	0.0
DRB1*11:01-DQB1*03:01	**97**	**133**	0.7	0.5	0.9	1.4×10^−2^	2.9×10^−1^	6.1	**53**	**42**	1.3	0.8	2.0	2.4×10^−1^	5.1×10^0^	0.1
DRB1*14:01-4-DQB1*05:031	**29**	**44**	0.7	0.4	1.0	7.6×10^−2^	1.6×10^0^	3.2	**19**	**10**	1.9	0.9	4.2	9.1×10^−2^	1.9×10^0^	0.0
DRB1*01:02-DQB1*05:01	**44**	**73**	0.6	0.4	0.9	6.3×10^−3^	1.3×10^−1^	7.5	**19**	**19**	1.0	0.5	1.9	1.0×10^0^	2.1×10^1^	0.1
DRB1*10:01-DQB1*05:01	**27**	**47**	0.6	0.4	0.9	1.9×10^−2^	3.9×10^−1^	5.5	**10**	**8**	1.3	0.5	3.2	6.3×10^−1^	1.3×10^1^	0.0
DRB1*04:05-DQB1*02:01	**16**	**31**	0.5	0.3	0.9	2.8×10^−2^	5.8×10^−1^	4.9	**10**	**5**	2.0	0.7	5.9	1.9×10^−1^	4.1×10^0^	0.5
DRB1*16:01-DQB1*05:02	**177**	**321**	0.5	0.4	0.6	2.6×10^−12^	5.4×10^−11^		**94**	**85**	1.1	0.8	1.5	4.7×10^−1^	9.8×10^0^	2.2
DRB1*15:02-DQB1*06:01	**10**	**37**	0.3	0.1	0.5	7.3×10^−5^	1.5×10^−3^	15.7	**11**	**8**	1.4	0.6	3.5	4.9×10^−1^	1.0×10^1^	0.1
**Others (<1%)**	**101**	**133**							**44**	**48**						
**Total**	**1600**	**1660**							**638**	**638**						

Legend: Transmission disequilibrium test (TDT); Transmitted (T); Not transmitted (NT); p value (P); p value corrected (Pc).

As expected, there was no significant difference between transmitted and un- transmitted haplotypes in healthy siblings (data not shown)

### Independence of the effect and mode of inheritance of positively associated haplotypes

To avoid confounding results due to over-transmission, the transmission of each positively associated DRB1*-DQB1* haplotype was assessed by analysing the segregation from parents who carried only one associated haplotype, and removing all parents who carried other associated haplotypes. Despite a small statistical significance after correction, the *15:01-*06:02 haplotype was considered “positively associated”, partly because it is widely regarded as a/the major genetic determinant in MS risk. The analysis determined the risk to offspring that was conferred by having one copy of each specifically associated haplotype, independently from the other associated haplotypes (effect independence); versus two copies of “neutral” haplotypes (dominant effect); and the risk conferred by two copies versus one copy (additive effect).

*13:03-*03:01. The independent effect of *13:03-*03:01 was assessed by examining segregation of the haplotype in various mating types from the genotype of both parents. Due to the relative rarity of this haplotype, there were only 16 families available for the analysis. Possible mating combinations are *13:03-*03:01/*X by *X/*X, *13:03-*03:01/*X by *13:03-*03:01/*X and *13:03-*03:01/*13:03-*03:01 by *13:03-*03:01/*X (*X denotes haplotypes other than *13:03-*03:01, *04:05-*03:01, *03:01-*02:01 and *15:01-*06:02). Only the haplotype *13:03-*03:01/*X in a *X/*X combination was found, i.e. twelve *13:03-*03:01/*X *vs* four *X/*X offspring (OR = 3.0, P = 4.6×10^−2^). This finding is consistent with an independent predisposing effect of this haplotype and with a dominant model of inheritance. Data are shown in Table 2.

**Table 2:** Inheritance of HLA-DRB1*13:03-DQB1*03:01 in the absence of the other predisposing haplotypes DRB1*03:01-DQB1*02:01, DRB1*15:01-DQB1*16:02, DRB1*04:05-DQB1*03:01.

Mating genotype	Offspring genotype	
	13/13 versus 13/X	13/X versus X/X
13/X by X/X	NA	12 *vs* 4 (OR 3.0; P = 4.6×10^−2^)
13/X by 13/X	0	0
13/13 by 13/X	0	NA
**Total (16 Families)**		**12 ** ***vs*** ** 4** (OR 3.0; P = 4.6×10^−2^)

Legend: HLA-DRB1*13:03DQB1*03:01 (13); all other haplotypes (X); p value (P). NA means that offspring genotypes are not possible with parental genotypes.

*04:05-*03:01. Similarly, inheritance of the *04:05-*03:01 haplotype was assessed in 52 families. There was no parent carrying two copies of *04:05-*03:01. Between the mating type *04:05-*03:01/*X by *X/*X (*X denotes haplotypes other than *04:05-*03:01, *13:03-*03:01, *15:01-*06:02 and *03:01-*02:01), there were 37 *04:05-*03:01/*X *vs* 13 *X/*X (OR = 2.8, P = 6.9×10^−4^). Between the mating type *04:05-*03:01/*X by *04:05-*03:01/*X there were 2 *04:05-*03:01/*X *vs* 0 *X/*X offspring. In total there were 39 *04:05-*03:01/*X *vs* 13 *X/*X (OR = 3.0, P = 3.1×10^−4^), supporting an independent predisposing effect of this haplotype, which is coherent with a dominant model of inheritance. Data are shown in Table 3.

**Table 3:** Inheritance of HLA-DRB1*04:05-DQB1*03:01 in the absence of the other predisposing haplotypes DRB1*03:01-DQB1* = 02:01, DRB1*13:03-DQB1*03:01, DRB1*15:01-DQB1*06:01.

Mating genotype	Offspring genotype	
	4/4 versus 4/X	4/X versus X/X
4/X by X/X	NA	37 *vs* 13 (OR = 2.8; P = 6.9×10^−4^)
4/X by 4/X	0 *vs* 2 (OR = 0; P = 1.6×10^−1^)	2 *vs* 0 (P = 1.6×10^−1^)
4/4 by 4/X	0 *vs* 0	NA
**Total (52 Families)**	**0 ** ***vs*** ** 2**	**39 ** ***vs*** ** 13 (OR = 3.0; P = 3.0**×**10^−4^)**

Legend: HLA-DRB1*04:05-DQB1*03:01 (4); all other haplotypes (X); p value (P). NA means that offspring genotypes are not possible with parental genotypes.

*03:01-*02:01. Inheritance of the *03:01-*02:01 haplotype was assessed after omitting all parents carrying one of the other positively associated haplotypes (i.e., *03:01-*02:01, *13:03-*03:01, *15:01-*06:02 and *04:05-*03:01). The analysis was carried out in 435 families. There were 153 *03:01-*02:01/*X *vs* 104 *X/*X (OR = 1.5, P = 2.2×10^−3^) offspring from the mating type *03:01-*02:01/*X by *X/*X genotype, 41 *03:01-*02:01/*X *vs* 13 *X/*X (OR = 3.2, P = 1.4×10^−4^) offspring from the mating type *03:01-*02:01/*X by *03:01-*02:01/*X parents. In total there were 194 *03:01-*02:01/*X *vs* 117 *X/*X (OR = 1.7, P = 1.3×10^−5^) offspring. From the mating type *03:01-* 02:01/*X by *03:01-*02:01/*X there were 58 *03:01-*02:01/*03:01-*02:01 *vs* 41 *03:01-*02:01/*X (OR = 1.4, P = 8.8×10^−2^) and 49 *03:01-*02:01/*03:01-*02:01 *vs* 17 *03:01-*02:01/*X (OR = 2.9, P = 8.2×10^−5^) offspring from the mating type *03:01-*02:01/*03:01-*02:01 by *03:01-*02:01/*X parents. In total, there were 107 *03:01-*02:01*/03:01-*02:01 *vs* 58 *03:01-*02:01/*X (OR = 1.8, P = 1.4×10^−4^) offspring. These results are consistent with an independent effect of the haplotype, with a dominant inheritance, and with an additive effect on risk, since *03:01*0201/*X individuals had a risk 1.7-fold higher over the *X/*X carriers, while a 1.8-fold risk was carried by the *03:01-*02:01/*03:01-*02:01 individuals over the *03:01-*02:01/*X subjects. Data are shown in Table 4.

**Table 4:** Inheritance of HLA-DRB1*03:01-DQB1*02:01 in the absence of the other predisposing haplotypes DRB1*13:03-DQB1*03:01, DRB1*15:01-DQB1*06:02, DRB1*04:05-DQB1*03:01.

Mating genotype	Offspring genotype	
	3/3 versus 3/X	3/X versus X/X
3/X by X/X	NA	153 *vs* 104 (OR = 1.5; P = 2.2×10^−3^)
3/X by 3/X	58 *vs* 41 (OR = 1.4; P = 8.8×10^−2^)	41 *vs* 13 (OR = 3.2; P = 1.4×10^−4^)
3/3 by 3/X	49 *vs* 17 (OR = 2.9; P = 8.5×10^−5^)	NA
**Total (435 Families)**	**107 ** ***vs*** ** 58 (OR = 1.8; P = 1.4**×**10^−4^)**	**194 ** ***vs*** ** 117 (OR = 1.7; P = 1.3**×**10^−5^)**

Legend: HLA-DRB1*03:01-DQB1*02:01 (3); all other haplotypes (X); p value (P). NA means that offspring genotypes are not possible with parental genotypes.

### Independence of the effect and mode of inheritance of negatively associated haplotypes

*16:01-*05:02. The independence of the effect of the negatively associated *16:01-*05:02 haplotype was assessed by examining its inheritance either with the inclusion of parents carrying one or more predisposing haplotypes, or without any of the predisposing haplotypes. We denoted the predisposing haplotype with a *X, but not the protective *15:02-*06:01 haplotype, and identified 428 available families. In total there were 165 *16:01- *05:02/*X *versus* 250 *X/*X (OR = 0.7, P = 3.0×10^−5^). Offspring carrying two copies of the *16:01-*05:02 haplotypes had 0.1-fold less risk of developing MS compared to heterozygous individuals (P = 1.1×10^−7^), which is consistent with an additive model. Data are shown in Table 5.

**Table 5:** Inheritance of HLA-DRB1*16:01-DQB1*05:02 in the absence of the negatively associated HLA-DRB1*15:02-DQB1*06:01.

Mating genotype	Offspring genotype	
	16/16 versus 16/X	16/X versus X/X
16/X by X/X	NA	132 *vs* 227 (OR = 0.6; P = 2.0×10^−2^)
16/X by 16/X	4 *vs* 33 (OR = 0.1; P = 1,9×10^−6^)	33 *vs* 23 (OR = 1.4; P = 1.8×10^−1^)
16/16 by 16/X	1 *vs* 8 (OR = 0.1; P = 2.0×10^−2^)	NA
**Total (428 Families)**	**5 ** ***vs*** ** 41 (OR = 0.1; P = 1.1**×**10^−7^)**	**165 ** ***vs*** ** 250 (OR = 0.7; P = 3.0**×**10^−5^)**

Legend: HLA-DRB1*16:01-DQB1*05:02 (16); X includes the positively associated DRB1*03:01-DQB1*02:01, DRB1*13:03-DQB1*03:01, DRB1*15:01-DQB1*06:02, DRB1*04:05-DQB1*03:01 and “neutral” haplotypes; p value (P). NA means that offspring genotypes are not possible with parental genotypes.

To avoid effects due to the over-transmission of positively associated haplotypes, the analysis was repeated for parents who were negative for all of the associated haplotypes, and the protective *15:02-*06:01 haplotype. Of the 108 families available for our analysis, there was 1 *16:01- *05:02/*16:01-*05:02 *vs* 17 *16:01- *05:02/*X (OR = 0.1, P = 1.6×10^−4^) and 52 *16:01- *05:02/*X *vs* 50 *X/*X (OR = 1.0, *X denotes haplotypes other than *13:03-*03:01, *15:01-*06:02,*04:05-*03:01,* 03:01-*02:01 and *15:02-*06:01). The independence of the haplotype effect was thus confirmed in homozygous individuals, since the presence of two copies of the *16:01-*05:02 haplotype significantly lowered the MS risk with respect to a single copy. Moreover, heterozygous individuals had risk similar to neutral haplotype homozygous carriers, which suggests a recessive inheritance mechanism of the *16:01-*05:02 haplotype. Data are shown in Table 6.

**Table 6:** Inheritance of HLA-DRB1*16:01-DQB1*05:02 (16) in the absence of the negatively associated HLA-DRB1*15:02-DQB1*06:0, the positively associated DRB1*03:01-DQB1*02:01, DRB1*13:03-DQB1*03:01, DRB1*15:01-DQB1*06:02 and DRB1*04:05-DQB1*03:01. X = “neutral” haplotypes.

Mating genotype	Offspring genotype	
	16/16 versus 16/X	16/X versus X/X
16/X by X/X	NA	40 *vs* 41 (OR = 1.0; P = 9.1×10^−1^)
16/X by 16/X	1 *vs* 12 (OR = 0.1; P = 2.3×10^−3^)	12 *vs* 9 (OR = 1.3; P = 5.1×10^−1^)
16/16 by 16/X	0 *vs* 5 (OR = 0; P = 2.5×10^−2^)	NA
**Total (108 Families)**	**1 ** ***vs*** ** 17 (OR = 0.1; P = 1.6**×**10^−4^)**	**52 ** ***vs*** ** 50 (OR = 1.0; P = 8.4**×**10^−1^)**

X = all other alleles “neutral” haplotypes.

Legend: HLA-DRB1*16:01-DQB1*05:02 (16); “neutral” haplotypes(X); p value (P). NA means that offspring genotypes are not possible with parental genotypes.

With the limitation of a small number of families, we repeated the analysis relative to the *15:02-*06:01 haplotype, as previously done for the *16:01-*05:02 haplotype. After omitting families carrying the protective *16:01-*05:02 haplotype, and including as *X also those families with the predisposing *13:03-*03:01, *15:01-*06:02, *04:05-*03:01 and *03:01-*02:01 haplotypes, only 28 families remained available for the analysis. There were six *15:02-*06:01/*X *vs* 22 *X/*X offspring (OR = 0.3, P = 2.5×10^−3^) from the mating type *15:02-*06:01/*X by *X/*X parents, consistent with a dominant model. Removal of the positively associated haplotypes left no parents available for analysis, thus it was not possible to confirm the independence effect. Data are reported in Table 7.

**Table 7:** The inheritance of HLA-DRB1*15:02-DQB1*06:01 (*15:02) in the absence of HLA-DRB1*16:01-DQB1*05:02.

Mating genotype	Offspring genotype	
	*15:02/*15:02 versus *15:02/X	*15:02/X versus X/X
*15:02/X by X/X	NA	6 *vs* 22 (OR = 0.3; P = 2.5×10^−3^)
*15:02/X by *15:02/X	0	0
*15:02/*15:02 by *15:02/X	0	NA
**Total (28 Families)**		**6 ** ***vs*** ** 22 (OR = 0.3; P = 2.5**×**10^−3^)**

Legend: HLA-DRB1*15:02-DQB1*06:01 (*15:02); X includes the positively associated DRB1*03:01-DQB1*02:01, DRB1*13:03-DQB1*03:01, DRB1*15:01-DQB1*06:02, DRB1*04:05-DQB1*03:01 and “neutral” haplotypes; p value (P). NA means that offspring genotypes are not possible with parental genotypes.

### Case and pseudocontrol analysis

A conditional logistic regression analysis was designed to identify genotypes associated with MS and to evaluate potential confounding interaction effects between haplotypes. Significant associations were identified through a backward stepwise approach, consecutively eliminating from the model the interactions that are not significant (P>5×10^−3^). The analysis revealed six significantly associated haplotypes. Potential data over-fitting problems, leading to alpha error rate increases, were excluded by performing 1000 random permutations within each matched set. The same model was obtained using forward selection from the null model. ORs of associated haplotypes relative to all non-associated ones, denoted as DRB1*X-DQB1*X, showed that four haplotypes increased the risk of MS: *04:05-*03:01 (OR = 2.6, 95% CI 1.8–3.6,P = 8.2×10^−8^); *13:03-*03:01 (OR = 3.2, 95% CI 1.7–6.0, P = 2.0×10^−4^); *15:01-*06:02 (OR = 2.2, 95% CI 1.3–3.8, P = 4.0×10^−3^); and *03:01- *02:01 (OR = 1.8, 95% CI 1.5–2.1, P = 8.7×10^−12^). Two haplotypes decreased risk: *16:01-*05:02 (OR = 0.7, 95% CI 0.6–0.9, P = 4.1×10^−4^) and *15:02-*06:01 (OR = 0.3, 95% CI 0.2–0.7, P = 4.0×10^−3^). The analysis showed no significant interaction between haplotypes. A subsequent conditional logistic regression analysis was performed with a backward stepwise approach to estimate the risk associated with homozygous genotypes, and heterozygous genotypes consisting of one predisposing or protecting haplotype combined with one unassociated haplotype. The combined risk of two different predisposing or protecting haplotypes was also estimated. The carriage of two predisposing or protective haplotypes increased or decreased MS risk according to a multiplicative model. The genotype ranking, in decreasing order of risk was: from *04:05-*03:01/*03:01-*02:01 (OR = 4.5, 95% CI 2.2–9.3, P = 4.3×10^−5^); *03:01-*02:01/*03:01-*02:01 (OR = 4.1, 95% CI 2.9–5.8, P = 2.5×10^−15^); and *16:01-*05:02/*16:01-*05:02 (OR = 0.2, 95% CI 0.1–0.5, P = 1.1×10^−3^). Heterozygous individuals exhibited the highest risk if they carried the *13:03-*03:01/*X (OR = 3.2, 95% CI 1.7–5.9, P = 2.6×10^−4^) genotype; followed by *04:05-*03:01/*X (OR = 2.8, 95% CI 1.9–4.0, P = 2.2×10^−8^); *15:01-*06:02/*X (OR = 2.2, 95% CI 1.3–3.8,P = 4.8×10^−3^); *03:01-*02:01/*X (OR = 1.40, 95% CI 1.2–1.7, P = 3.9×10^−4^); and *16:01-*06:01/*X (OR = 0.4, 95% CI 0.2–0.7, P = 4.8×10^−3^) genotypes. The risk remained high for individuals with genotypes combining one predisposing and one protective haplotype, although P values were not significant and 95% CIs were large, due to the scarce presence of such combinations. Data are presented in Table 8.

**Table 8:** HLA DRB1*-DQB1* genotypes from 943 multiple sclerosis patients and 362 healthy siblings, plus 2829 pseudocontrols obtained from healthy parents of the 943 trio families.

HLA DRB1*-DQB1* genotype	P	OR	95% CI	
03:01-02:01/03:01-02:01	2.5×10^−15^	4.1	2.9	5.8
16:01-05:02/16:01-05:02	1.1×10^−3^	0.2	0.1	0.5
04:05-03:01/X	2.2×10^−8^	2.8	1.9	4.0
13:03-03:01/X	2.6×10^−4^	3.2	1.7	5.9
15:01-06:02/X	4.8×10^−3^	2.2	1.3	3.8
03:01-02:01/X	3.9×10^−4^	1.4	1.2	1.7
16:01-05:02/X	1.1×10^−2^	0.8	0.6-	0.9
15:02-06:01/X	4.8×10^−3^	0.4	0.2	0.7
04:05-03:01/03:01-02:01	4.3×10^−5^	4.5	2.2	9.3
04:05-03:01/16:01-05:02	8.6×10^−3^	4.5	2.2	9.3
13:03-03:01/03:01-02:01	1.5×10^−2^	6.7	1.4	31.2
13:03-03:01/16:01-05:02	4.4×10^−2^	9.4	1.1	84.0
15:01-06:02/03:01-02:01	3.9×10^−2^	3.7	0.5	7.7

Legend: X = all not associated haplotypes. P values threshold is set at 0.005.

Results were confirmed by relative predispositional effect (RPE) analysis (data not shown).

### Sequence alignment and structural analysis

Since the two protective *16:01 and *15:02 alleles are serologically “split” from the DR2 as well as the predisposing *15:01 allele, we focused functional analysis on these three alleles and evaluated sequences similarity by multiple alignment of DRB1*15:01, *16:01 and *15:02. The variable residue at position 86, belonging to Pocket-1 of the DRB1 chain, is the only difference between the protective (Gly86 in the *16:01 and in the *15:02 alleles) and predisposing (Val86 in the *15:01 allele) DR2 splits. Similarly, we examined sequence similarity of DQB1 chain for the predisposing *06:02 allele and the protective *05:02 and *06:01 alleles. Also in this case, the DQ partners of different DR2 presented a variation that is clearly linked to a/the predisposing/protective allele typology. More precisely, this variable residue is located at position 38, under Pocket-9, with Ala38 in the predisposing DQB1*06:02 allele, and Val38 in the two protective DQB1 *05:02 and *06:01 alleles. Sequence alignments are reported in Table 9 and 10.

**Table 9:** Multiple Sequence alignments of the predisposing and protective alleles considered in the study, with only variable amino acid residues reported, for the three DRB1 alleles (*15:02, *16:01, *15:01) belonging to serological group DR2.

*Position*	*CLASS*	9	10	11	12	13	47	57	67	70	71	74	77	86
DRB1*15:02	**Protective**	W	Q	P	K	R	F	D	I	Q	A	A	T	***G***
DRB1*16:01	**Protective**	W	Q	P	K	R	Y	D	F	D	R	A	T	***G***
DRB1*15:01	**Predisposing**	W	Q	P	K	R	F	D	I	Q	A	A	T	***V***
*Pockets*		P9		P6		P4	P7	P9	P7	P4		P4		P1
						P6					P7			

The pockets to which resides belong to are indicated in the last two lines.

**Table 10:** Multiple Sequence alignments of the predisposing and protective alleles considered in the study, with only variable amino acid residues reported, for the three DQB1 alleles (*06:01,*05:02,*06:02).

*Position*	*CLASS*	9	13	14	26	28	30	37	38	55	57	74	75	77	84	85	86
DQB1*06:01	**Protective**	L	A	M	Y	T	Y	D	**V**	R	D	E	L	T	E	V	A
DQB1*05:02	**Protective**	Y	G	L	G	T	H	Y	**V**	R	S	S	V	R	E	V	A
DQB1*06:02	**Predisposing**	F	G	M	L	T	Y	Y	**A**	R	D	E	L	T	E	V	A
*Pockets*		P6	P4		P4	P4	P6	P9			P9	P4				P1	

## Discussion

The strong association between HLA-DRB1-DQB1 loci and MS has been established across many populations, with consistent findings indicating that predisposition is carried by the *15:01-* 06:02 haplotype in all populations of North-European ancestry [4], while in Israel [11] and in Mediterranean [8]–[10] populations, predisposition to the disease is carried by different DRB1* variants. In the light of these findings it is interesting to note that a recent genome-wide association study confirmed the *15:01 allele as the strongest genetic determinant in MS and, after conditioning to the *15:01, an association with the DRB1*03:01 and the DQB1*02:01 alleles emerged. After further conditioning, a third secondary association with the *13:03 allele was evident [4].

Several studies indicated that the presence of alleles confers resistance to the disease and modulates the permissive effect of the *15:01 allele, thus suggesting that the autoimmune response may be lowered or cancelled by the co-presence of both susceptible and protective alleles. Indeed, two copies of the *15:01 allele determined the highest risk [13], while the *15/*14 genotype considerably lowered the risk of the disease [13]–[16]. However, the extended DRB1*-DQB1*haplotype has not been taken into account in these studies [13]–[16], despite findings that support the effect of both DRB1*15:01 and DQB1*16:02 alleles [17], [18] in MS risk.

In Sardinia, an Italian island having a very high prevalence of MS [21], [22] and a peculiar genetic background [23], a heterogeneous HLA association with MS has been reported [24]. We re-analysed the risk carried from HLA class II variants, and specifically determined by both DRB1* and DQB1* alleles (DRB1*- DQB1* haplotype) in a Sardinian MS family sample more than twice as large as in our previous study [24]. Our intent was to confirm the haplotype association, to establish the independence of the associated haplotypes and assess the risk due to the genotype.

First, the association was evaluated in 943 trio Sardinian families. Using a highly conservative correction of P values, TDT analysis confirmed that in Sardinia HLA-related MS susceptibility is associated with *13:03-*03:01 (OR = 2.9), *04:05-*03:01 (OR = 2.4) and *03:01-*02:01 (OR = 2.1) haplotypes, while the previously reported over-transmission of a DRB1*15:01-DQB1*06:02 haplotype was no longer found after correction for multiple comparisons, so the previously reported over-transmission of the DRB1*04:05-DQB1*03:02 haplotype was not confirmed. In the second step, the independence of the effect of the various associated haplotypes was assessed by inheritance analysis of each associated haplotype, which may be transmitted from parents to affected offspring, removing other associated haplotypes. Independence of the positively associated *13:01-*03:01, *04:05-*03:01 and *03:01-*02:01 haplotypes was thus established. The presence of one copy of the haplotype *vs* two copies of neutral haplotypes was sufficient to increase the risk, thus showing that all predisposing haplotypes are inherited according to a dominant model. An additive effect on risk was observed relative to the *03:01-*02:01 haplotype. In offspring carrying one copy of this haplotype had a risk that was 1.7 times higher than for those carrying neutral haplotypes (that are all non associated haplotypes); while homozygous individuals had a 1.8-fold higher risk than heterozygous ones.

TDT analysis showed that two haplotypes, namely *16:01-*05:02 (OR = 0.5) and *15:02-*06:01 (OR = 0.3), were under-transmitted. To remove the possibility of an over-transmission bias, the haplotype inheritance from parents was studied by removing all positively associated haplotypes. The protective effect of *16:01-*05:02 was confirmed in homozygous *vs* heterozygous patients (OR = 0.1), as occurs in a recessive model; while heterozygous individuals showed the same risk of their offspring carrying two copies of the neutral haplotypes (OR = 1.0). The relative rarity of the *15:02-*06:01 haplotype precluded the possibility of determining, by analysis alone, a real effect for haplotype under- transmission in the absence of susceptible haplotypes. Thus, the independence of the effect has not been established. However, findings obtained using the RPE method, indicate that the negative association of *15:02-*06:01 haplotype with MS was not due to a displacement effect driven by the positively associated haplotypes. It is important to observe that, the negative associations were not detected in previous RPE analyses [24]. This apparent contradiction is likely due to both the high frequency of the *16:01-*05:02 haplotype and the low frequency of the *15:02-*16:01 haplotype in the general Sardinian population (18.8% and 1.6%, respectively, in 2756 chromosomes from healthy individuals, data not shown), which would require an adequately large sample size [25] for an affordable analysis.

Positive and negative associations were further confirmed by examining the risk as determined by genotype. Logistic regression analysis of associated haplotypes in comparison to all non-associated ones, showed that four haplotypes significantly increased the risk of MS: *04:05-*03:01 (OR = 2.6); *13:03-*03:01 (OR = 3.2); *15:01-*06:02 (OR = 2.2); and *03:01- *02:01 (OR = 1.8); while two haplotypes decreased the risk, *16:01-*05:02 (OR = 0.7) and *15:02-*06:01 (OR = 0.3). Contrary to some analyses in other populations, where a recessive effect of the *03:01 allele has been reported [17], [21], [26], but in agreement with data from Canadian population [14]; our analysis consistently confirmed the dominant effect of this allele. Within the logistic risk framework a dose effect was also observed in “at risk” homozygous individuals, as in compound heterozygous individuals, according to an additive model. Thus, the highest risk was conferred by *04:05-*0301/*03:01-*02:01 (OR = 4.5); followed by *03:01-*02:01/*03:01-*02:01 (OR = 4.1). In contrast, while *16:01-*05:02/*16:01-*05:02 decreased the risk (OR = 0.2), one copy alone of *16:01-*05:02 was not sufficient to confer protection in genotypes that combine this haplotype with a predisposing one. Consistent with the findings from haplotype inheritance analyses, the effect of MS associated DRB1-DQB1 genotypes delineates a model constituted by a dominantly acting susceptibility gene contained on, or near to, the *04:05-*03:01, *13:03-*03:01, *03:01-*02:01 haplotypes, in conjunction with the absence of a protective gene required for the maintenance of peripheral tolerance. Another example of allelic interactions acting in human disease comes from type 1 diabetes, where the greater relative risk is carried by the DRB1*03 and *04 when compared with DRB1*03 or *04 homozygous [27]. The same effect appears in celiac disease, where the risk is increased in carriers of the DQA1*05-DQB1*02 alleles either in *trans* or in *cis* [28].

In the particular genetic make-up of Sardinian population, the *15:01 allele is virtually absent; while the *16:01 is common [23], inverting the proportion of the allele frequencies as observed in North-European populations [4]. For the two alleles *16:01 and *15:02, we found a negative association with MS in the Sardinian families, and observed that both are “splits” of the serological group DR2, as is the susceptibility *15:01 allele [29]. A direct comparison of DRB1 sequence of *15:01, *16:01 and *15:02 was thus affected by multiple alignment. The variable residue at position 86 of the DRB1 protein chain is the only one that differentiated between the protective and the predisposing DR2 alleles [30]. Position 86 is one of the residues defining Pocket-1, which is fundamental for the correct positioning of the antigen peptide in the binding site [31]. As already described [31], when a Valine is present (as in the *15:01) the interaction with small aliphatic residues is favoured, while when a Glycine is present (as for *1502 and *1601), binding of large aromatic residues of the peptide is favoured. Similarly, we compared the DQB1 chains for the predisposing *06:02 allele and the protective *05:02 and *06:01 alleles. The DQ partners of different DR2 alleles presented a striking residue variation, this time at position 38, which mirrored the same kind of classification observed for DRB1. In particular, Ala38 is present in the predisposing *06:02, while Val38 is observed in both the protective alleles, *05:02 and *06:01. Position 38 is located under Pocket-9, composed by residues 30, 37 and 57. The Alanine to Valine change may determine some modifications to the respective positions of the residue in Pocket-9, thus impacting upon the binding mode. In particular, the main effect is likely due to steric properties, because the Valine side chains occupy a larger volume than Alanine side chains. Note that the DQB1 Pocket-9 residue Asp57 has a protective effect against some kinds of type 1 diabetes [32], [33]. A molecular explanation might be that, in the presence of Asp57, a saline bridge is formed between Asp57 and a conserved Arg79, locking Arg79 so that it is no longer free to interact with negatively charged P9 peptide residues (as in the case of non-Asp57 residue).

A role for the Val86 residue in MS susceptibility has been reported in Swedish [33] and Australian [34] populations. The differences in residues of the DRB1 and their cognate DQB1 chains observed in Sardinian MS patients could account for the different strength of association that characterises the disease-predisposing *15:01 allele and the pair of disease-protective *16:01 and *15:02 alleles. This implies a high degree of specificity for encephalitogenic molecules, as demonstrated for the DRB1 chain Val86 residue [30]. The high LD across the *15:01-*06:02 haplotype make it problematic to dissect whether the primary association is with the DRB1* or with the DQB1 molecules. Findings from African-American MS patients seem to support the idea that DRB1* is the primary driver of the association [35], but other data indicate that both DRB1 and DQB1 molecules are fundamental players in determining individuals' MS risk [19]. The present data from a Sardinian population suggest that both DRB1 and DQB1 chains contribute to MS predisposition, highlighting the importance of the amino acid residue in binding pockets and surrounding areas, particularly concerning their steric and the electrostatic properties. However, these findings do not explain why several phylogenetically different [36], [37] DRB1-DQB1 haplotypes harbour genetic susceptibility to MS in Sardinian and in other populations. One can hypothesise that each of these permissive haplotypes presents different myelin basic protein epitopes, or different autoantigens, in a sophisticated specialized way, but detailed functional studies from transgenic animal MS models would be required for proof.

In conclusion, the findings presented here demonstrate that independent, but associated, DRB1*-DQB1* haplotypes confer susceptibility and resistance to MS in the MS-prone Sardinian population. We suggest that similarities and differences in specific residues of the DRB1 and DQB1 chains may contribute to genetic predisposition for MS. In this regard, studies from genetically selected and homogeneous populations may contribute to a better understanding of the role of both DRB1* and DQB1* alleles in the MS autoimmune process.

## Methods

### Patients

We examined 943 MS families consisting of one affected sibling and both healthy parents (trio), and 362 healthy siblings (one from each family) of patients coming from the same families. Of these, 448 families have already been reported [24] while 495 constituted an independent sample. All patients participating in the study attended the MS Clinic at University of Cagliari (Italy). The study was conducted in accordance with the Helsinki Declaration and approved by University of Cagliari/ASL8 (Italy) ethics committee. All subjects gave informed written consent.

All patients included in the study met MS criteria [38], [39]. The study cohort included healthy subjects and Sardinian MS patients, many of them had a Sardinian ancestry of three generations or more. The sample included in the study was representative of the total Sardinian MS population consisting in about half of the estimated Sardinian population with MS (the actual Sardinian population is about 1 million and 550,000 inhabitants). Subjects included in the study came from each Sardinian province, and were present in proportion to the number of inhabitants of each province.

### Genotyping

Typing of the HLA-DRB1* and -DQB1* loci was performed as described elsewhere [24]. Briefly, the polymorphic second exon of the HLA-DRB1* and -DQB1* genes was amplified and the amplified products were dot-blot analysed using primers and SSO probes as described previously [24]. A total of 3191 individuals were thus typed.

### Statistical analysis

#### Transmission disequilibrium test analysis

Transmission of haplotypes was assessed by the TDTphase program (version 2.403) for the 943 trio families. The TDT counts the number of time a haplotype is transmitted from heterozygous parents to offspring. The χ^2^ distribution was used to assess significance. Only haplotypes present in at least 1% of sample were considered, grouping together the others. A highly conservative Bonferroni correction to P values (P_c_) for the total number of haplotypes investigated (N = 21) was applied.

#### The Relative predispositional effect method

The RPE method [40] was used to confirm the results obtained using conditional logistic regression analysis. It sequentially compares allele frequencies in patients and pseudocontrols to determine their predisposition, protective, or neutral effects relative to each other.

#### Inheritance analysis of predisposing/protective DRB1-DQB1 haplotypes

To examine the independence of each associated haplotype's effect, segregation of the haplotypes was assessed in various mating types from the DRB1*-DQB1* parental genotype [16]. Transmission of each positively associated haplotype was examined in the offspring of parents carrying the specific haplotype (1 copy or two copies); and of parents carrying 1 copy or 0 copies of the haplotype, after removing all the other associated haplotypes. The unassociated haplotypes were included in the analysis and denoted as *X. Alternatively, inheritance of each negatively associated haplotype was assessed from parents carrying, or missing, all the positively associated haplotypes. ORs were obtained by comparing the number of offspring inheriting two copies of the haplotype on analysis (A/A) versus those carrying one copy (A/*X), and comparing the number of offspring who inherited one copy (A/*X) versus those carrying 0 copies (*X/*X) obtained from the various parental mating types.

#### Case and pseudocontrol analysis

Genetic data of the 943 trio families, consisting of parents with a single affected offspring, were available for use. The control population consists of 362 healthy siblings and 2829 pseudocontrols obtained from the trio families; from each trio three pseudo-controls were derived from the three other possible combinations of haplotypes that could be transmitted from the parents. Each case was matched to three pseudo-controls [41]. A conditional logistic regression analysis was designed to identify haplotypes associated to MS and to evaluate potential confounding and interaction effects. At first, the haplotypes having a occurrence of at least 10 were considered as independent variables and inserted in the logistic model as “indicator variables”; haplotypes with an occurrence lower than 10 were grouped and inserted in the model as a reference group. Significant associations were identified through a backward stepwise approach, consecutively eliminating from the model the interactions and the haplotypes considered not significant (P>5×10^−3^).

Possible data over-fitting effects, which would have increased alpha error rates, were managed by performing 1000 random permutation within each matched set. A subsequent conditional logistic regression analysis was performed with a backward stepwise approach to produce estimates of the MS risk associated with homozygous genotypes, and heterozygous genotypes consisting of one predisposing or protecting haplotype combined with one non associated haplotype referred to as “X”. The combined risk of two different predisposing or protecting haplotypes was also estimated. In order to adjust for multiple testing the alpha level was set at 0.005.

#### Sequences alignment and structural analysis

Complete sequences of predisposing and protective haplotypes where obtained from the IMGT/HLA database (http://www.ebi.ac.uk/imgt/hla/) [42]. Sequences were aligned with the online tool Clustalw (http://www.ebi.ac.uk/Tools/clustalw2) [43] and binding pockets were then identified based on the annotation from Stern et al. [44] for DRB1* alleles. Based on the crystal structures of DQB1*06:02 from Siebold et al. [45], and of DQB1 03:02 from Lee et al. [46], the DQB1* pockets were defined as the set of residues within a 6 Å radius sphere centred on the peptide beta carbon atoms.
